# Vestibular Rehabilitation Outcomes in the Elderly with Chronic Vestibular Dysfunction

**DOI:** 10.5812/ircmj.3507

**Published:** 2012-11-15

**Authors:** Arash Bayat, Akram Pourbakht, Nader Saki, Zuraida Zainun, Soheila Nikakhlagh, Golshan Mirmomeni

**Affiliations:** 1 Department of Audiology, Tehran University of Medical Sciences, Tehran, Iran; 2 Department of Otolaryngology, Ahwaz Jundishapour University of Medical Sciences, Ahwaz, Iran; 3 School of Health Sciences, Universiti Sains Malaysia (USM), Malaysia

**Keywords:** Vestibular Rehabilitation, Chronic Vestibular Dysfunction, Dizziness, Handicap Inventory

## Abstract

**Background:**

Chronic vestibular dysfunction is a frustrating problem in the elderly and can have a tremendous impact on their life, but only a few studies are available. Vestibular rehabilitation therapy (VRT) is an important therapeutic option for the neuro-otologist in treating patients with significant balance deficits.

**Objectives:**

The purpose of this study was to assess the effect of vestibular rehabilitation on dizziness in elderly patients with chronic vestibular dysfunction.

**Materials and Methods:**

A total of 33 patients older than 60 years with chronic vestibular dysfunction were studied. Clinical and objective vestibular tests including videonystagmography (VNG) and dizziness handicap inventory (DHI) were carried out at their first visit, 2 weeks, and 8 weeks post-VRT. The VRT exercises were performed according to Cawthorne and Cooksey protocols.

**Results:**

Oculomotor assessments were within normal limits in all patients. Nineteen patients (57.57%) showed abnormal canal paralysis on caloric testing which at follow-up sessions; CP values were decreased remarkably after VRT exercises. We found a significant improvement between pre-VRT and post-VRT total DHI scores (P < 0.001). This improvement was most prominent in functional subscore.

**Conclusions:**

Our study demonstrated that VRT is an effective therapeutic method for elderly patients with chronic vestibular dysfunction.

## 1. Background

Dizziness is a common and troubling symptom in the elderly population (1, 2). Approximately 20% of the elderly living in a community report having dizziness severe enough to interfere with their routine activities (3). It is estimated that in one third of people over the age of 40, vestibular dysfunction with dizziness the risk of falls increases by 12 times compared to normal people (4, 5). Furthermore, vestibular dysfunction can lead to loss of independence, seriously affecting the quality of life (6, 7) and even death in older people (8). Considering all those impacts, clinicians should take special considerations in management of this symptom. It has been demonstrated that vestibular rehabilitation therapy (VRT) is a safe and effective method for most individuals with vestibular or balance disorders (9, 10). The general goal of VRT is to reduce the vertiginous symptoms and to promote patient's functional balance, physical mobility and overall activity level (11, 12).

To measure the effects of the vestibular exercises on dizziness recovery, we performed a combination of subjective and objective tests including videonystagmography (VNG) and Dizziness Handicap Inventory (DHI) scale. VNG using electro-oculography to record eye movements while stimulating the vestibular system. The results of VNG can be used to determine the site of lesion, which is beneficial in providing further diagnosis and management (13). DHI is a reliable questionnaire related to balance-derived handicaps known to quantify the impact of dizziness on daily activities (14, 15). DHI can also be used for measuring how symptoms affect quality of life (14).

Vestibular rehabilitation in Iran usually offers to a small minority of vertiginous patients, after a lengthy process of referral to specialist clinics. It is often the last treatment option when no other remedy can be suggested. The delay in providing rehabilitation may lead to vicious cycle, whereby patient's physical, cognitive and psychosocial activities are restricted because of provoking disequilibrium and vertigo (16, 17). There is only limited evidence evaluating the effect of VRT on balance function and confidence in the elderly patients with chronic vestibular dysfunction.

## 2. Objectives

This study attempts to determine the effectiveness of vestibular rehabilitation in the elderly with chronic vestibular dysfunction.

## 3. Materials and Methods

We recruited 33 patients with age over 60 years old with chronic decompensated peripheral vestibular dysfunction. All subjects underwent full neurologic and otologic assessments. Patients with cervical, visual problems, cognitive, orthopedic, or neurologic disorders; patients having fluctuating and intermittent vertigo, and duration of symptoms less than 4 months; and patients with bilateral decompensated vestibular disorder were excluded from the study. All vestibular suppressing medications were discontinued three days before the commencement of the study.

Clinical and objective vestibular tests were carried out at their first visit, 2 weeks, and 8 weeks post-VRT. VNG examination (ICS Chartr 200 VNG, GN Otometrics) consisted of recording the spontaneous nystagmus, followed by oculomotor (random saccade, gaze, eye tracking, optokinetic nystagmus), positioning, positional, and bithermal caloric tests. Maximum velocity of the slow-phase component of nystagmus was analyzed for canal paresis (CP) and directional preponderance (DP) indexes. CP or DP indexes ≥ 25% were considered normal.

We used DHI scale to quantify the effects of the vestibular exercises on symptom's recovery. The DHI consists of 25 questions organized in three different dimensions: physical (7 questions), emotional (9 questions) and functional (9 questions). Patients answered “yes” (4 points), “sometimes” (2 points) and “no” (0 points). The total score ranged from 0 to 100 points.

The vestibular rehabilitation exercises used in the current study were primarily taken from protocols established by Cawthorne and Cooksey. These exercises were administered twice a week for two months, followed by an assimilation of repetitions at home. The objective of this protocol was to improve static and dynamic balance function, with an enhancement of gaze stabilization. This protocol was selected because of its easy administration and group performance possibility. We considered follow-up sessions to offer guidance, if necessary.

Clinical efficacy of the treatment was evaluated by VNG testing and DHI score prior to VRT, 2 and 8 weeks post-VRT. These pre- and post-VRT differences were analyzed in relation to gender and affected ear, too.

Data were analyzed using the SPSS statistical package, version 16. Descriptive statistics revealed that the data were normally distributed and appropriate for parametric analysis methods. To test changes in DHI scores across different trials, the repeated measures analyses of variance (between consecutive trials) were performed. A significance level of 0.05 was used for all the analyses. This study was approved by the local ethics committee of Ahwaz Jundishapour University of Medical Sciences, and informed consent forms were obtained from all participants.

## 4. Results

The subjects were included 12 men (36.36 %), aged 61-71 years old, with mean age of 59.2 (± 3.08) years old; and 21 women (63.64 %), with ages between 61 and 74 years old, mean age of 66.80 (± 3.84) years old. In the first visit, no spontaneous nystagmus was detected via VNG recordings. Oculomotor assessments were within normal limits in all patients. Nineteen patients (57.57%) showed abnormal CP on caloric testing lateralized to right (n=12) or left (n=7) ears; and 13 subjects (42.43%) had normal test results. At follow-up sessions, CP values were decreased remarkably after 8 weeks VRT, so that 8 other subjects revealed normal caloric responses.

**Figure 1:**
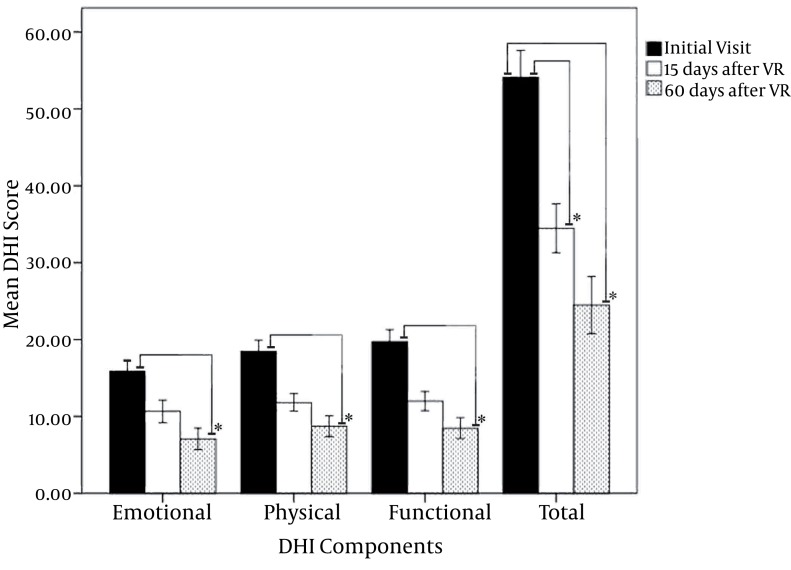
Means of the total Dizziness handicapped inventory (DHI) scores and its subcomponents before and after vestibular rehabilitation (VR) therapy; *: statistically significant)

The mean total DHI score was improvement about 19.64 and 29.64 points at 2 and 8 weeks post-VRT, respectively. Total DHI scores also demonstrated improvements after VRT compared to before VRT examinations (P < 0.001). We found no significant differences between men and women or those with right- or left-sided lesions (P > 0.05). In the analyses of pre- and post-VRT mean DHI points, a decrease and a consequent improvement were observed in all the aspects (emotional, functional and physical). This improvement was most prominent in functional subscore( Figure 1 ).

## 5. Discussion

In the present study, we observed rapid improvements in symptoms, dizziness-related disability, and postural stability in patients with chronic decompensated vestibular deficit after short-term VRT program. Similar to other studies (18, 19) this study also has shown that VRT has a positive effect on the symptoms of chronic vestibular failure. VRT is an exercise-based approach designed to maximize central nervous system compensation in vestibular pathology (20-22). VRT will accelerate and improve central compensation via the mechanisms of habituation training, which enhances adaptation of the vestibulo-ocular and vestibule-spinal reflexes as well as substitution.

The current investigation used the DHI, a well validated measure of balance handicap, to look at the outcome of VRT. A significant statistical improvement was observed between total DHI scores before and after rehabilitation exercises (2 weeks and 8 weeks). The DHI subscores illustrated that a VRT program changes patient’s perceptions related to physical, emotional and functional impacts of their dizziness. The decrease in the severity of dizziness-induced disability with rehabilitation exercises enables patients to live independently and improves their quality of life (8).

Our current investigation did not show any statistically difference between total DHI improvement score and gender. This result is in consistent with Jung et al. (10), and supports that in the elderly gender is not a significant factor in predicting the outcome of rehabilitation. For individual total DHI scores, a change of at least 18 points regarded clinically significant (15). Using this criterion, 23 patients (69.69 %) showed a dramatic improvement causing no restriction in their lifestyles. An additional 10 patients (30.31 %) showed partial improvement means their symptoms have been made some restriction in their activities.

Our results demonstrated that there is no relationship between the length of treatment and eventual outcome. Longer duration of treatment is not necessarily better, and patients should be discharged once they cease to make any remarkable progress (23). Although some authors believe that elderly patients require extra treatment time compared to younger patients and full vestibular compensation is never attained, but Jung et al. (10) and Bittar et al. (24) noted that age is not a significant factor in response rate to vestibular exercises .Our data also showed that elderly patients respond favorably to VRT.

Bittar et al. (24) indicated that the time at which VRT is recommended is crucial for success. Unsatisfactory responses may be due to VRT being conducted at the wrong moment, when the patient has not yet reached a favorable clinical state. In agreement with Jung et al. (14), we believe that an appropriate VRT program may help to minimize the effects of age-related deterioration of the vestibular system and its psychological impact. Our results indicated that vestibular rehabilitation is a useful therapeutic approach to promote a more efficient recovery from chronic vestibular dysfunction in elderly patients and age factor makes no difference on therapy outcome. It is a low-cost, short and safe treatment modality which can be widely employed in outpatients without the need of sophisticated gadgets.
